# Apelin/APJ: Another Player in the Cancer Biology Network

**DOI:** 10.3390/ijms26072986

**Published:** 2025-03-25

**Authors:** Laura Naldi, Alessandro Peri, Benedetta Fibbi

**Affiliations:** 1 “Pituitary Diseases and Sodium Alterations” Unit, AOU Careggi, 50139 Florence, Italy; laura.naldi@unifi.it (L.N.); benedetta.fibbi@unifi.it (B.F.); 2 Endocrinology, Department of Experimental and Clinical Biomedical Sciences “Mario Serio”, University of Florence, 50139 Florence, Italy

**Keywords:** apelin, APJ, cancer, hallmarks of cancer, chemoresistance

## Abstract

The apelinergic system exerts multiple biological activities in human pathologies, including cancer. Overactivation of apelin/APJ, which has been detected in many malignant tumors, and the strong correlation with progression-free and overall survival, suggested the role of an oncogene for the apelin gene. Emerging evidence sheds new light on the effects of apelin on cellular functions and homeostasis in cancer cells and supports a direct role for this pathway on different hallmarks of cancer: “sustaining proliferative signaling”, “resisting cell death”, “activating invasion and metastasis”, “inducing/accessing vasculature”, “reprogramming cellular metabolism”, “avoiding immune destruction” and “tumor-promoting inflammation”, and “enabling replicative immortality”. This article reviews the currently available literature on the intracellular processes regulated by apelin/APJ, focusing on those pathways correlated with tumor development and progression. Furthermore, the association between the activity of the apelinergic axis and the resistance of cancer cells to oncologic treatments (chemotherapy, immunotherapy, radiation) suggests apelin/APJ as a possible target to potentiate traditional therapies, as well as to develop diagnostic and prognostic applications. This issue will be also covered in the review.

## 1. Introduction

The neuropeptide apelin is the endogenous ligand for the receptor APJ, a seven-transmembrane, G protein-coupled receptor, which has high affinity with the angiotensin II receptor type 1a [1,2,3]. Human apelin is encoded by the *APLN* gene, which encodes a 77-amino-acid precursor peptide (pre-pro-apelin) [4]. The enzymatic hydrolysis of pre-pro-apelin originates several biologically active peptides, which share the APJ-binding C-terminal sequence [5]. Different apelin isoforms (apelin-12, -13, -15, -16, -17, -19, -28, -31, -36, and -55, which are identified based on their numbers of amino acids) display peculiar receptor binding affinity [5], with apelin-13 representing the most effective activator of APJ [6]. Elabela/APELA/Toddler is a micropeptide encoded by the *APLN* gene and recognized as the second endogenous ligand for APJ [7]. Its expression appears to depend on developmental stage, with the highest expression in the inner part of the blastocyst and a progressive downregulation during differentiation [8].

**Figure 1:**
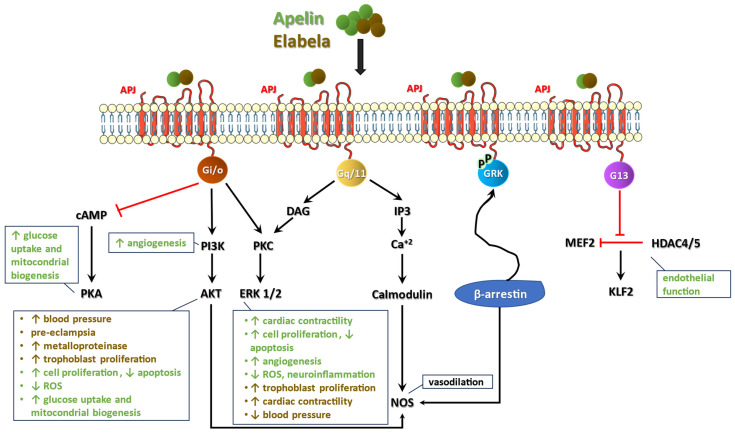
Intracellular pathways and cellular functions modulated by the APJ system. By interacting with G proteins, the binding of the endogenous ligand (apelin or Elabela) to APJ is able to promote the following: the inhibition of cAMP generation and protein kinase A (PKA) and activation of phospho-inositide 3-kinase (PI3K)/AKT through Gi/o; the activation of protein kinase C (PKC)-dependent extracellular signal-regulated kinase 1/2 (ERK1/2) through Gi/o or Gq/11; the initiation of the intracellular release of Ca^2+^ by Gq/11 and inositol 1,4,5-triphosphate (IP3) synthesis; the autophosphorylation of APJ through G protein-coupled receptor kinase (GRK) and initiation of the β-arrestin-mediated cascade; the activation of G13 and inactivation of histone deacetylases (HDAC) type 4 and 5, determining the activation of myocyte enhancer factor-2 (MEF2) and expression of MEF2 target gene Kruppel-like factor 2 (KLF2). Both AKT activation and increase of intracellular Ca^2+^ and β-arrestin induce nitric oxide synthase (NOS). The up arrows indicate upregulation. The down arrows indicate downregulation. ROS: reactive oxygen species.

APJ interaction with G proteins leads to the modulation of many signaling pathways and cellular functions after ligand binding (Figure 1): (i) the activation of the phospho-inositide 3-kinase (PI3K)/Akt and protein kinase C (PKC)/extracellular signal-regulated kinase 1/2 (ERK 1/2) pathways; (ii) the downregulation of protein kinase A (PKA) by inhibiting adenylate cyclase and cAMP production; (iii) the upregulation of phospholipase C beta (PLCβ), which triggers the generation of diacylglycerol (DAG) and inositol 1,4,5-triphosphate (IP3), thus leading to the initiation of the PKC cascade and the intracellular release of Ca^2+^ respectively [9,10]. Furthermore, apelin binding induces the autophosphorylation of APJ through G protein-coupled receptor kinase (GRK) and starts G protein-independent signaling mediated by β-arrestin [10,11]. Finally, APJ has been shown to activate G13 in human umbilical vein endothelial cells, leading to the modulation of endothelial function [12]. Both apelin and APJ are highly conserved among species and widely expressed in tissues, including brain, spinal cord, heart, lung, placenta, endocrine glands, bone marrow, skeletal and smooth muscles, gastrointestinal and urinary organs, and adipose tissue [13]. Activation of APJ signaling modulates several physiological processes. Specifically, it is involved in the regulation of cell proliferation and migration, apoptosis, glucose metabolism, mitochondrial biogenesis, and energetic homeostasis [12]. It promotes brown adipocyte differentiation and the browning of white fat, angiogenesis, neuroinflammation, oxidative stress, nitric oxide release and vasodilation (by relaxing the smooth muscle cells of blood vessels), cardiac contractility, and heart rate [12]. Moreover, pituitary secretion, food intake, body fluid homeostasis, glucose metabolism, and the neuroendocrine stress response are regulated by APJ-dependent intracellular pathways, as well as immune and gastrointestinal functions [12]. The great heterogeneity of apelin/APJ’s biological actions is mainly explained by the interaction of APJ with different G proteins and intracellular pathways, according to cell type, microenvironment, and apelin isoforms [12], as reported in Figure 1.

In the last two decades, a large amount of evidence has highlighted the role of the apelin/APJ system in carcinogenesis and tumor progression. The common denominator of apelin/APJ activation in cancer is oxidative stress, which is induced by hypoxia (generated by the hypermorphosis of cancer cells) and upregulates *APLN* expression through reactive oxygen species-dependent hypoxia inducible factors (HIFs) [14,15]. As a matter of fact, apelin and APJ expression in tissues and circulating levels of apelin have been reported to correlate with prognosis in different cancers [16]. Moreover, *in vitro* and in vivo results have shown the role of the apelinergic system in affecting tumor growth, angiogenesis, cell metabolism, immune microenvironment, and metastasization [16]. Although the landscape of the biological role of apelin/APJ in cancer is complex and clinical trials using apelin and its antagonists are lacking, this axis is a promising, novel therapeutic target for several malignancies. This review will focus on the currently available literature about the cellular processes and molecular pathways involved in apelin/APJ-dependent regulation of cancer biology. Furthermore, the relationship between the apelinergic system and resistance to anticancer treatments will be discussed, and the possible role of APJ antagonism in oncology will be explored.

## 2. The Apelin/APJ System in Cancer

### 2.1. Role of Apelin/APJ as a Prognostic Marker in Different Human Cancers

An upregulation of the apelin/APJ system was observed in several malignant tumors and strongly correlated with cancer progression and high mortality, making apelin emerge as a potential oncogene. In cervical squamous cell carcinoma and endocervical adenocarcinoma tissues, apelin expression was significantly higher than in normal tissues and positively associated with advanced clinicopathologic features, poor therapy outcomes, and short survival [17,18,19].

In a cohort of 142 breast cancer patients, apelin expression was detected in 59.2% of tumors and significantly correlated with tumor size and stage, microvessel density, lymph node metastasis, and worse disease-free and overall survival [20]. Increased apelin levels were also found to be an independent predictor of HER-2/neu expression and a more aggressive breast cancer phenotype [21]. TCGA pan-cancer analysis revealed that the apelin gene is most consistently upregulated in hepatocellular carcinoma (HCC) among all cancer types. Specifically, it is overexpressed in 90% of HBV-associated and in 82% of non-alcoholic fatty liver disease (NAFLD)-associated HCC, where it is highly induced compared to adjacent normal tissues. Although apelin mRNA did not correlate with TNM staging, it was found to be an independent prognostic factor of shorter overall survival in three cohorts of HCC patients [22]. In a study of 288 patients with curatively resected HCC, it was demonstrated that high APJ expression was significantly associated with the presence of intrahepatic metastasis and early recurrence, as well as shorter progression-free (PFS) and overall (OS) survival. In addition, in multivariate analysis, high APJ expression was an independent predictor of shorter PFS (hazard ratio 1.49; 95% confidence interval 1.08–2.05, *p* = 0.016) [23]. Furthermore, sustained high apelin expression levels in advanced T stages (T3 and T4) suggested that liver tumors characterized by high apelin/APJ signaling were likely to progress rapidly beyond the T2 stage [24]. In gastroesophageal carcinoma, the tissue level of apelin was higher than in healthy controls [25], and a lower 3000-day survival rate was observed in patients with high apelin expression in esophageal cancer tissues compared to those with low and medium expression [26]. Apelin expression status in tissue is also closely associated with more advanced disease and poorer outcomes in gastric cancer [27], non-small cell lung carcinoma (NSCLC) [28], and cholangiocarcinoma [29] subjects. Apelin expression of prostatic adenocarcinoma was higher than in normal prostate and high-grade intraepithelial neoplasia [30]. Apelin upregulation more frequently occurred in prostate cancer tissues with advanced tumor stage and metastasis and was associated with a shorter biochemical recurrence-free survival [31]. Increased APJ expression significantly correlated with a decreased median OS of 14.7 months in patients with high-grade serous ovarian cancer [32].

Comparison of colorectal cancer and colorectal non-tumor tissues showed increased levels of apelin mRNA and protein, with a tumor/non-tumor ratio of 2.5 and 1.68, respectively. Similar results were also observed for APJ (ratio of 4.24 for mRNA, 7.98 for protein). Analysis of clinicopathological parameters revealed higher levels of serum apelin in patients with more advanced TNM stages, and lymph node and distant metastasis. Conversely, no correlation was observed with apelin concentration in tumors. In this view, circulating apelin has been proposed as a diagnostic and prognostic marker [33]. In a series of 42 gliomas paired with their paracancerous tissues, APJ was upregulated in glioma tissues compared to the adjacent ones. Based on the evidence that patients with higher APJ levels had advanced stage and worse prognosis, the authors suggested APJ as a carcinogenic gene able to accelerate the development of glioma [34].

Together with intratumoral apelin expression, a higher tumor stage, a higher probability of distant metastasis, and a shorter PFS and OS were also associated with serum apelin levels in muscle-invasive bladder cancer patients [35], head and neck cancer [36,37], breast cancer [38], multiple myeloma [39], lung adenocarcinoma [40], and glioblastoma [41,42]. The correlation between circulating levels of apelin and clinical outcomes in cancer patients was further investigated by a clinical study conducted in a cohort of 95 patients affected by different cancers (34% lung cancer, 24% gastrointestinal cancer, 18% breast cancer, 9% gynecological cancer, and 15% prostate cancer), treated with one or more lines of chemotherapy, surgery, and/or radiotherapy. Cancer patients had higher apelin levels than healthy subjects (532.5 ± 223.4 vs. 231 ± 47.1 pg/mL) and apelin concentrations were closely correlated with disease stage. Moreover, subjects with progressive diseases had significantly increased apelin levels at baseline compared to non-progressor patients. Univariate followed by multivariate Cox proportional hazards regression analysis showed that apelin predicted cancer progression independently of other potential confounders [43].

Significantly higher levels of Elabela have been shown in the serum of patients affected by chronic lymphocytic leukemia than in control patients [44], whereas increased Elabela gene expression has been reported in glioblastoma cells and associated with poor prognosis [45].

### 2.2. Apelin/APJ Affects the Hallmarks of Cancer: Evidence from In Vitro and In Vivo Models

**Figure 2:**
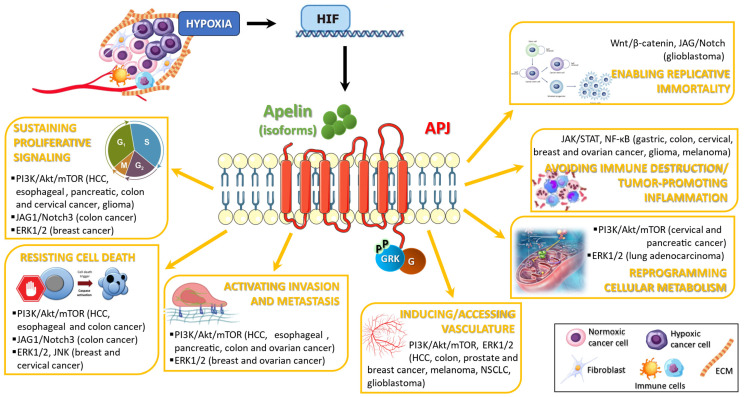
Modulation and biological effects of the apelin/APJ system in cancer. Hypoxia, generated by the hypermorphosis of cancer cells, upregulates apelin expression by inducing oxidative stress and dependent hypoxia inducible factor (HIF). The overactivation of the apelinergic system in tumor tissue directly contributes to cancer development and progression through the sustainment of several hallmarks of cancer: sustaining proliferative signaling, resisting cell death, activating invasion and metastasis, inducing/accessing vasculature, reprogramming cellular metabolism, avoiding immune destruction and tumor-promoting inflammation, and enabling replicative immortality. The apelin/APJ effect on each hallmark is driven in different malignancies by the modulation of several intracellular pathways. ECM: extracellular matrix.

The hallmarks of cancer are defined as functional capabilities acquired by cells as products of multistep processes, and crucial for their transition from normalcy to neoplastic growth states [46]. Among those, the apelin/APJ system has been reported to directly interact with “sustaining proliferative signaling”, “resisting cell death”, “activating invasion and metastasis”, “inducing/accessing vasculature”, “reprogramming cellular metabolism”, “avoiding immune destruction” and “tumor-promoting inflammation”, and “enabling replicative immortality” (Figure 2).

#### 2.2.1. Sustaining Proliferative Signaling

Cancer cell proliferation is the basis of tumorigenesis and metastasis. In recent years, apelin has been identified as a mitogenic signal transducer through APJ and associated with a variety of cancers [5].

The PI3K/Akt/mTOR pathway responds to the availability of nutrients, hormones, and growth factors and has been shown to play a significant role in tumor cell growth and proliferation. Its activation occurs in the early stages of tumor development and correlates with poor prognosis and therapeutic resistance in various human cancers [8,47]. Accordingly, the targeting of the PI3K/Akt/mTOR pathway disrupts the development of multiple tumors [48]. The PI3K/Akt pathway plays a significant role in the development and progression of HCC. The effect of apelin/APJ in promoting HCC through the activation of PI3K/Akt was demonstrated *in vitro* by the increased expression of phospho-glycogen synthase kinase 3β (p-GSK3β) and cyclin D1 induced by apelin via APJ, which in turn promoted cell proliferation and accelerated G1/S progression. Accordingly, inhibition of apelin was found to downregulate the apelin-PI3K/Akt cascade and to suppress the growth of liver cancer cells, while apelin knockdown reduced HCC xenograft growth in mice [22]. Wang et al. showed that apelin mediates the proliferation of esophageal cancer cells via PI3K/mTOR [26]. A similar apelin-stimulated upregulation of PI3K/Akt signaling and increased proliferation was reported in pancreatic [49] and cervical cancer cells [17]. The facilitating role of apelin in cervical cancer carcinogenesis and progression was evidenced by the fact that apelin silencing inhibited the proliferation of CaSki and SiHa cells and abrogated the phosphorylation of PI3K, AKT, and mTOR, as confirmed by a tumor transplantation experiment. Additionally, opposite results were determined by apelin overexpression and reverted by the APJ antagonist ML221 and the PI3K inhibitor LY294002 [17].

In a colon adenocarcinoma cell line, a study by Picault et al. showed that apelin enhanced cell proliferation through adenylyl cyclase inhibition and Akt phosphorylation [50]. Additionally, apelin induced the proliferation of LS180 cells via upregulation of Notch3, resulting in the activation of JAG1/Notch3 signaling [51]. APJ-stimulated proliferation of glioma cells was also mediated by the activation of Akt, which is induced in this cell model by the downregulation of the transcription factor Nuclear Factor of Activated T cells 5 (NFAT5) [34].

The involvement of ERK1/2 in apelin-induced proliferation was revealed in MCF-7 breast cancer cells, where the transcription of hormone-dependent breast cancer amplified 1 (AIB1) promoted the activation of ERK and the expression of cyclin D1 [52] and lung adenocarcinoma A549 [40]. The increased cell growth induced by apelin-13 was reverted by the presence of exogenous estrogen. Hence, APJ signaling was proposed to prevent tumor growth under conditions of estrogen starvation and to represent a possible new therapeutic target in endocrine resistance of breast cancer cells [53]. On the other hand, apelin could eliminate the pro-stimulating effect of estradiol on ovarian cancer cell proliferation through the crosstalk between APJ, the estrogen receptor alpha, and insulin-like growth factor-1 receptor [54].

Accordingly, with increased Elabela levels detected in ovarian cancer cells, the disruption of its expression in these cell lines suppressed cell growth and cell cycle progression in a p53-dependent manner [55].

#### 2.2.2. Resisting Cell Death

The apoptotic intrinsic pathway is regulated by the Bcl-2 family, whose members (Bcl-2, Bax, and Bak) homo-oligomerize when activated, regulate outer mitochondrial membrane permeability, and subsequently induce irreversible caspase activation and apoptotic cell death [56]. Several studies had shown that the apelin/APJ pathway exerts anti-apoptotic functions by acting on different apoptosis-related proteins, specifically enhancing Bcl-2 levels and decreasing Bax and cleaved caspase-3 levels [57,58,59,60]. The anti-apoptotic effects of the apelinergic system are mainly mediated by the PI3K/Akt, MAPK/ERK1/2, and JNK signaling pathways, even in cancer models. As a matter of fact, PI3K/Akt/mTOR activation driven by apelin overexpression is able to inhibit apoptosis of HCC [22], esophageal [26], and colon adenocarcinoma [50] cells. Specifically, apelin decreased caspase-3 activity and poly ADP ribose polymerase (PARP) proteolysis in LoVo cells [50], and in a xenograft model glioblastoma [42]. Conversely, the high-survival phenotype of cervical cancer cells is characterized by MAPK activation [18].

#### 2.2.3. Activating Invasion and Metastasis

Existing evidence shows that the overexpression of apelin/APJ in malignant cells is related not only to the acceleration of tumor growth, but also to metastasization. In mouse and human melanoma cell lines, apelin overexpression had no impact on cell proliferation, whereas it significantly induced migration and promoted a more invasive phenotype in a 3D collagen invasion assay [61]. In a metastatic *in vivo* model, where cancer cells were injected intravenously, apelin significantly increased both the number and size of lung metastases [61]. The role of apelin/APJ in promoting cell migration and invasion has been also recognized in breast cancer via the ERK1/2/AIB1 pathway and the secretion of MMP-1 [52], and in esophageal, HCC, and pancreatic cancer cells through PI3K/mTOR signaling [26,49,62]. In gastric cancer, apelin has been suggested to prompt tumor invasiveness through the upregulation of several cytokines (including IL-1, IL6, MMP-1, MMP-9, and BMP-2) known to be correlated with tumor invasiveness and metastasis [27]. The expression of apelin and APJ have been closely linked to the progression of colon cancer due to affecting the PI3K/AKT pathway and inducing cell migration and invasion through bleb formation and activation of proteolytic enzymes that digest the extracellular matrix (ECM) (MMP-2, MMP-9, MT1-MMP) [63]. Similarly, it has been proposed that the apelin/APJ axis regulates lung metastasis of breast cancer cells through the upregulation of some key metastasis-inducing factors, including MMP-2, MMP-3, MMP-9, osteopontin, focal adhesion kinase (FAK), platelet-derived growth factor (PDGF), and stromal cell-derived factor 1 chemokine receptor alpha 4 (SDF-1α/CXCR4) [64].

In addition to increasing the migration and invasion of cervical carcinoma cells, apelin upregulated the expression of Snail, Vimentin, and N-cadherin and reduced E-cadherin, thus promoting epithelial–mesenchymal transition (EMT), through which epithelial cells acquire motile and invasive characteristics [17].

In ovarian cancer, APJ is specifically overexpressed in metastatic tissues compared to primary tumors. This correlation is the clinical expression of a more pro-metastatic phenotype *in vitro* (including proliferation, cell adhesion to various ECM molecules, anoikis resistance, migration, and invasion) and an increased metastasization rate *in vivo*, due to the activation of the STAT3, ERK, and AKT pathways downstream of APJ [32]. Consistently with apelin/APJ’s ability to confer an aggressive phenotype to cancer cells, invading glioblastoma cells expressed high levels of APJ, which correlated with increased expression of genes involved in tumor cell invasion like MMP-2 or BAI1/3, and experimentally downregulating *APLN* expression in orthotopic *in vivo* models led to increased tumor invasiveness [65,66]. Accordingly, treatment with apelin-13 advanced migration and invasion of lung adenocarcinoma cells via the p21-activated kinase-1 (PAK1)-cofilin signaling pathway [67]. The transformation of ECM has a critical role in the proliferation and metastasis of osteosarcoma [68]. As a matter of fact, HIF-1 promotes ECM remodeling under hypoxic conditions by inducing in fibroblasts the expression of enzymes involved in collagen crosslinking, stabilization, and deposition [69]. Among those, procollagen-lysine 2-oxoglutarate 5-dioxygenase (PLOD) 2 is critical for tumor cell motility [70], and its expression is increased by apelin in osteosarcoma tissues, thereby promoting cancer cell migration and metastasis. Accordingly, an inhibitor of PLOD2 could suppress apelin-induced promotion of osteosarcoma cell migration, and the development of lung metastasis was inhibited in mice injected with apelin knockdown cells [71]. Levels of apelin and PLOD2 expression were also positively associated with tumor staging in patients [71].

#### 2.2.4. Inducing/Accessing Vasculature

Tumor angiogenesis is defined as the proliferation of a network of blood vessels which penetrates the tumor mass, supplying nutrients and oxygen, removing waste products, and thus promoting cancer cell growth [72]. Hypoxia is considered a major trigger for the initiation of angiogenesis through the activation of angiogenic genes, such as VEGF and apelin, by HIF [72]. It has been reported that VEGF signaling positively regulated APJ expression in tumor blood vessels, and that both APJ [73] and apelin inhibition were able to reshape the tumor microenvironment (TME) and suppress angiogenesis and growth [74]. The role of the apelinergic system in directly activating tumor neovascularization is exerted through its paracrine effect on promoting the formation of the new blood vessels required for cancer growth [75]. In a colorectal cancer model, intestinal progenitor cell expansion promoted blood vessel growth in a VEGFA-independent manner, which involved the upregulation of endothelial apelin expression. The migration of venous endothelial cells towards the stem/progenitor cell zone stabilized intestinal crypt vessels and prevented hypoxia, due to the oxygen-dependent metabolism of transformed stem/progenitor cells during pathological expansion. The importance of apelin in the maintenance of this balance is evidenced by the association between its deficiency and crypt hypoxia, and reduced proliferation and survival of intestinal progenitor cells [76]. In HCC, the first evidence of the involvement of the apelinergic system in angiogenesis was the correlation of high APJ expression with microvascular infiltration [23]. Most recently, single-cell sequencing localized an overexpression of both apelin and APJ in vascular endothelial cells of liver tissue, and spatial transcriptomics confirmed that endothelial populations with high APJ scores were enriched within the tumor. As counterproof, apelin silencing in endothelial cell lines reduced angiogenesis; endothelial proliferation; expression of ANG2, KLF2, and VEGFA; and ERK1/2 phosphorylation. Furthermore, the stemness of endothelial cells with high apelin/APJ expression was enhanced, as well as the expression of TGF-β, oxidative stress, and PI3K/Akt- related pathways [77]. The enhanced apelin pathway triggered downstream PI3K/Akt signaling to promote endothelial carcinogenesis during HCC development, thus highlighting the importance of apelin in cell communication inside the TME [24]. The apelin/APJ axis was further demonstrated to induce the morphological and functional maturation of blood vessels in mouse melanoma, human prostate cancer xenografts [78], and NSCLC models [28]. In *in vivo* mammary and breast cancer mouse models, apelin blockage inhibited tumor growth through the reduction of capillary leakage and tissue hypoxia, the maintenance of pericyte coverage, and the deregulation of VEGF-related angiogenetic pathways [74]. A dramatic upregulation of apelin and APJ mRNA expression was detected in glioblastoma-associated microvasculature, where a mutant APJ ligand was able to prevent tumor angiogenesis [66]. The central role of apelinergic signaling in neoplastic vascularization of glioblastoma was supported by a large amount of evidence [79,80,81], including the increase in vascular apelin and APJ gene expression concomitantly with a switch from an invasive to an angiogenic histopathological phenotype [65]. Consistently, apelin was found to be upregulated in the tumor neovasculature as compared to cancer-free control areas, whereas the orthotopic implantation of glioblastoma cells into apelin knockout mice resulted in a significantly lower angiogenesis than in wild-type animals. Therefore, the authors concluded that intratumoral endothelial cells are a major source of apelin in glioblastoma and that autocrine apelin/APJ signaling in endothelium is a key driver of tumor angiogenesis [65].

Besides its pro-angiogenic effect, apelin/APJ has been reported to induce the growth of lymphatic vessels. Apelin promoted the migration and capillary-like tube formation of lymphatic endothelial cells and increased their 3D growth. Furthermore, its overexpression in mouse melanoma cells stimulated tumor growth, intratumoral lymphangiogenesis, and lymph node metastasization *in vivo*, as well as increased blood and lymph vessel densities inside lung metastases [61].

#### 2.2.5. Reprogramming Cellular Metabolism

Glycolysis is a metabolic pathway used by cancer cells to generate energy for rapid cell proliferation. Consistent with evidence that its inhibition contributes to delayed tumor progression [82,83,84], the activation of glycolysis induced by apelin in non-cancer models [85,86] suggested a potential role for the apelin/APJ system in promoting tumor growth and progression even through a direct effect on this metabolic pathway. PI3K/Akt/mTOR signaling is an essential regulator of glycolysis [87]. Their close association during tumorigenesis [88] is confirmed by Akt inhibitors’ ability to reduce cervical cancer progression by mediating mTOR signaling and glycolysis [89]. In a cervical cancer model, apelin-induced activation of PI3K/Akt/mTOR improved glucose uptake (which was conversely abolished by the PI3K inhibitor LY294002), which was at least in part responsible for the stimulatory effect of apelin/APJ on cell proliferation and migration [17]. A similar apelin-dependent induction of glycolysis was also reported in pancreatic tumors [49].

Autophagy is a catabolic process by which the cell transports damaged components to lysosomes for digestion and degradation, in order to prevent the accumulation of waste products and toxins and provide nutrients during starvation [90]. Although autophagy is crucial to inhibit tumorigenesis in normal cells by removing carcinogenic elements, tumor cells intimately interact with their microenvironment and activate autophagy [91], which plays a major role in cancer progression by promoting cell proliferation and migration and by inhibiting cell apoptosis [92,93,94,95,96,97,98]. Overexpressed apelin/APJ has been related to activation of autophagy in cancer. In lung adenocarcinoma, apelin-13 induced autophagy through ERK1/2 activation [40]. This mechanism was strictly dependent on APJ, which promoted beclin-1 expression via HIF-1α [99]. The same authors demonstrated that the PAK1/cofilin-dependent effect of apelin on migration [67] was mediated by autophagy [99]. The discovery of the apelin–autophagy–cofilin pathway provides new markers to support the clinical diagnosis of lung adenocarcinoma and novel targets for treatment.

The HMGA1 protein is a transcriptional enhancer that induces oncogene expression by interacting with DNA and proteins [100,101], thus determining metabolic alterations that contribute to tumorigenesis through aerobic glycolysis and fatty acid synthesis [102,103]. The biological activity of HMGA1 is highly regulated by post-translational modifications including glutamine deamination [104], which is enhanced by apelin to stabilize the expression of HMGA1, thus promoting lipid metabolism and cell growth of lung cancer cells [105]. APJ silencing in apelin-overexpressing lung cancer cells did not completely inhibit apelin-mediated cell proliferation.

#### 2.2.6. Avoiding Immune Destruction and Tumor-Promoting Inflammation

The majority of cancers (90–95%) are associated with chronic inflammation, and tumor-associated inflammation has been determined to play a pivotal role in cancer by affecting almost every stage: cancer development, metastasization, drug resistance and cancer recurrence by inducing genomic instability, self-renewal of cancer stem-like cells, and angiogenesis [106]. Through their interaction with cellular (pro-inflammatory cells, intrinsic immune cells, stromal cells) and non-cellular (immune checkpoint factors and pro-inflammatory cytokines produced by intrinsic immune cells and the tumor) components, cancer cells activate different oncogenic pathways and improve immune escape. In other words, the interference of tumor-associated inflammation with tissue homeostasis is determined by the accumulation of innate (monocytes, natural killer cells, macrophages—the latter the only immune cells resident in tissues) and adaptive (T- and B-lymphocytes) immune cells in response to aberrant signals from the tumor [107,108]. However, their tolerogenic functions are hijacked by inflammatory mediators (interleukins such as IL-1, IL-6, IL-8, and IL-23, and chemokines such as CXCL1, CXCL2, CXCL5, CXCL8, and CXCR2) produced by cancer cells through the activation of multiple signaling pathways, including Janus kinase/signal transducers and activators of transcription (JAK/STAT) and nuclear factor kappa B (NF-κB), which leads to local immunosuppression and suppresses anti-tumor immunity [109].

The complex role of immunity in cancer passes through the balancing of CD4+ T-lymphocyte polarization into different subgroups, and the Th1 or Th2 helper T cells, which in turn induce macrophage differentiation in M1 or M2 subtypes, respectively [110]. Specifically, Th2 cells promote tumor progression through the secretion of cytokines IL-4, IL-6, IL-10, and IL-13 to activate tumor-associated M2 macrophages, which drive tumor proliferation and migration in both primary and metastatic sites by contributing to basal membrane disruption and accumulation, angiogenesis, leukocyte recruitment, and immunosuppression [110]. Increased TGF-β released by Th17 cells and T-regulatory cells also attenuates the immune response and has been associated with malignant conversion and progression in breast, gastric, endometrial, and ovarian cancer, in gliomas, and in melanomas [111]. Conversely, Th1 cells secrete cytokines that induce M1 tumor-associated macrophages and cytotoxic CD8+ T cells and promote tumor cell destruction through recruitment of leukocytes and phagocytosis of cancer cells [110].

*APLN* transcription is upregulated by inflammatory mediators and increased expression was reported, for instance, in intestinal tissue during inflammation [112]. Murine monocytes and macrophages express APJ, which mediated the anti-inflammatory effects of apelin by regulating the expression of inflammatory cytokines and chemokines [113]. The presence of APJ in T-lymphocytes was first revealed in 1998, when it was identified as a co-receptor for HIV entry into T cells [114,115]. Subsequently, the functionality of apelin/APJ in T- and B-lymphocytes was demonstrated by its involvement in the regulation of the adaptive immune system by suppressing cytokine production from mouse spleen cells in response to T cell receptor/CD3 cross-linking [116]. Most recently, APJ was identified as an essential gene for immunotherapy in cancer through the modulation of interferon γ signaling [117], which may exert both anti-tumor [118] and pro-tumor [119,120] effects with an overall outcome dependent on signal intensity, TME, and specificity. For example, the negative regulator of immune response PD-L1 is tightly regulated by IFN-γ in melanoma cells, thus contributing to their acquired immune resistance [121,122]. In cultured macrophages, apelin downregulated macrophage inflammatory protein (MIP)-1α, monocyte chemoattractant protein (MCP)-1, IL-6, and TNF-α [113], whereas it suppressed TLR4 (which is involved in the maturation of DCs as well as the differentiation of Th1 cells) [123] and NOD-like receptor family pyrin domain-containing 3 (NLRP3)-mediated inflammatory responses in a sepsis rat model [124]. The immunomodulatory effects of apelin are also exerted by targeting non-immune cells, as evidenced by the apelin-13-dependent decrease of pro-inflammatory factors inducible nitric oxide synthase and IL-6, and increase of immunosuppressive factors arginase-1 and IL-10 in a microglia cell line [125].

In cervical cancer, high expression levels of apelin were positively associated with the abundance of Th2 cells (which are associated with poor prognosis in various types of cancer) [126], while negatively correlated with infiltrating B cells (which have a positive prognostic effect on many cancer types) [127], thus suggesting an apelin/APJ role in promoting tumor-associated inflammation and immunosuppression [18]. The observation that apelin released from a head and neck squamous cell carcinoma cell line caused M2-type differentiation by decreasing IL1β expression further confirmed its contribution to creating an immunosuppressive environment that stimulates tumor progression [128]. In a mouse implantation model of mammary carcinoma, the reduction in tumor vessel density and tumor volume upon apelin depletion was associated with decreased myeloid-derived suppressor cells and increased natural killer cells [74]. A gene cluster analysis for APJ-coregulated genes in glioblastoma also indicated a role for apelinergic signaling in lymphocyte-mediated and natural killer-mediated immunity [129].

According to their cytotoxic function on cancer cells, marked accumulation of intra-tumoral CD8+ T cells correlates with better prognosis [130,131,132]. Infiltration of circulating CD8+ T cells into tumors is governed by adhesion molecules and chemokines expressed by endothelial cells [133]. In a xenograft model of colon cancer and chronic lymphocytic leukemia, Hu et al. showed that apelin upregulated the endothelial expression of CCL8 [134], a chemoattract protein involved in the recruitment of resting and activated T cells as well as other immune cells into tissues [135,136,137]. Consequently, CD8+ and CD4+ T cells accumulated more in the central areas of the tumors, whereas the distribution of macrophages and regulatory T cells throughout the tissues was not different in the presence or absence of apelin [134].

#### 2.2.7. Enabling Replicative Immortality

Cancer stem cells are a small group of cancer cells with the properties of normal stem cells, such as self-renewal and pluripotency, but also characterized by enhanced ability to initiate tumor growth, proliferate, invade, migrate, and resist therapeutics [138].

Apelin and APJ are known to be expressed in the developing mesoderm [66,139,140,141], and increasing evidence shows APJ expression in pluripotent stem cells, such as hematopoietic stem cells [142,143]. In several cancers, apelin might play a role in mediating the differentiation of mesenchymal stem cells into cancer stem cells, whose self-renewal has been facilitated by activating the Wnt/β-catenin and Jagged/Notch signaling pathways [64]. As well as in glioblastoma tumor cells, APJ expression was found in different subpopulations of stem cells (neural progenitor-like cells—NPCs, oligodendrocyte progenitor-like cells—OPCs, mesenchymal progenitor-like cells—MESs, reactive astrocytic cells), whose maintenance is supported by APJ activation [42].

Since Elabela expression has been detected in glioblastoma samples [45] and localized into the brain stem cell niches in nestin-positive cells [144], it could be involved in gliomagenesis as a mitotic factor for neoplastic tumor initiating nestin-positive cells. Moreover, apelin acts as a key regulator in maintaining glioblastoma stem cell spheroids, promoting the self-renewal of stem cells. Accordingly, the APJ antagonist MM54 was able to inhibit tumor growth [42,145].

## 3. Apelin/APJ and Drug Resistance in Cancer

### 3.1. Main Mechanisms of Drug Resistance in Cancer: The Interplay of the Apelin/APJ System

Chemoresistance, the ability of cancer cells to resist chemotherapeutic drugs, is a complex mechanism which represents a significant challenge in the search for an effective anticancer therapy. This ability of tumor cells can be attributed to a wide variety of processes, which depend on tumor heterogeneity, a high drug influx that reduces efficacy, the development of multidrug resistance, and the alteration of signaling pathways involved in the response to therapy [146,147].

One of the involved mechanisms is the presence of mutations that alter the processes of proliferation and apoptosis. Mechanisms of DNA damage repair play a crucial role in preserving genome stability by fixing errors or damage caused by physiological processes or environmental factors, including oxidative stress, radiation, chemicals, or mistakes during DNA replication. Different mechanisms of repair have developed, such as mismatch repair, base excision repair, nucleotide excision repair, and double strand break repair, to address different types of DNA damage. The impairment of repair systems determines the increase in the mutational burden, thus potentially triggering the development of cancer [148,149]. Acquired mutations let cancer cells proliferate uncontrollably and, consequently, evade the apoptotic signals triggered by chemotherapeutic agents. This can be caused by an increased activation of pro-apoptotic pathways, including NF-κB, PI3K/Akt, and MAPK/ERK [150], or the overexpression of anti-apoptotic factors (such as Bcl-2, Bcl-xL, and surviving)/downregulation of pro-apoptotic molecules (such as Bax, Bak, and caspase-3) [151]. In this scenario, APJ-dependent activation of PI3K/Akt promotes cell survival and reduces the effectiveness of chemo-induced apoptosis [64,152]. The apelin–APJ interaction also triggers the ERK/MAPK pathway, which is essential for cell proliferation and differentiation [153]. Similarly, it has been shown *in vitro* that apelin treatment protected cells from H_2_O_2_-mediated oxidative stress [154], promoted the expression of anti-apoptotic proteins (i.e., Bcl-2), and inhibited the expression of the pro-apoptotic proteins Bax and caspase-3 [154,155]. On the other hand, apelin silencing inhibited cell proliferation by downregulating the PI3K/AKT/mTOR pathway and the Bcl-2 protein family, and promoted apoptosis mediated by Bax and caspase-3 [156].

EMT has also been linked to increased chemoresistance. Activation of Snail, Slug, Twist, and Zeb1/2, transcription factors associated with EMT, has been associated with increased DNA repair capacity and upregulation of chemotherapeutic drug efflux transporters [157]. By activating repair systems such as nucleotide excision repair, base excision repair, and mismatch repair, cancer cells are able to survive the cytotoxic effects of chemotherapeutics [151]. Moreover, apelin may affect gene expression through epigenetic mechanisms, such as DNA methylation and histone modification [158]. Hu et al. demonstrated that apelin and Snail are highly expressed in breast cancer and might interact in the initiation and progression of carcinogenesis. Furthermore, their expression is positively associated with lymph node metastases and TNM staging, thus correlating with a poor prognosis [159].

Another well-studied mechanism of drug resistance is the overexpression of ATP-binding cassette (ABC) transporters, such as P-glycoprotein and drug resistance-associated proteins. Through these pumps, drugs which entered the cytoplasm are excreted into the extracellular environment, leading to a reduction in their cytotoxic effect on tumor cells. Overexpression of ABC transporters observed in several tumor types has been associated with an unfavorable outcome of chemotherapy [160,161]. The relationship between apelin and ABC transporters was demonstrated in an *in vitro* study. Specifically, apelin-13 has been shown to increase ABCA1 protein levels and promote cholesterol efflux from THP-1 macrophage-derived foam cells, through the activation of PKCα signaling [162]. A positive correlation between apelin and ABCA1 levels was also identified *in vivo* [163]. Beyond cellular components, the TME is able to influence the response to chemotherapy. Complex interactions of cancer cells with stromal cells or the extracellular matrix may influence their sensitivity to chemotherapy [164]. It has been demonstrated that apelin expression is elevated in the hypoxic conditions commonly found in the TME. HIFs activated by low oxygen levels interact with apelin signaling to regulate hypoxia-responsive non-coding RNAs (ncRNAs), which play a crucial role in angiogenesis by affecting genes involved in VEGF signaling [165]. Among stromal cells, cancer-associated fibroblasts (CAFs) are highly related to drug resistance in cancer. Tumor-derived IL-1β and constitutive IL-1 receptor-associated kinase 4 activated the NF-κB signaling pathway in CAFs and pancreatic cancer cells, whose sensitivity to gemcitabine was significantly attenuated [166], whereas CAFs-derived IL-6 promoted resistance to cisplatin via the JAK/STAT3 pathway in esophageal squamous cell carcinoma [167]. Additionally, CAFs promoted EMT and resistance to cisplatin in NSC cells via the TGF-β/IL-6 axis, and, in turn, cisplatin treatment increased the secretion of TGF-β in cancer cells, thereby leading to the amplification of CAFs activation [168].

The clinical application of immunotherapy is revolutionizing cancer treatment, although its therapeutic efficacy is still limited due to the immunosuppressive microenvironment inside tumors [169]. In this view, a large body of evidence suggests that, beyond the clearance of cancer cells, ideal treatments should include the disruption of tumor-induced immunosuppression by targeting tumor-associated inflammation and reactivating anti-tumor T cells. Tumor-associated macrophages are carriers of immune checkpoint ligands (PD-L1 or CTLA-4 ligand), whose overexpression determines the inhibition of the CD8+ T-lymphocytes’ response and the effect of immunotherapy [170]. Similarly, myeloid-derived suppressor cells avoid the anticancer activity of immunotherapies by negatively regulating the expression of immune checkpoint molecules [171]. Recent research revealed that apelin interacts with immune cells within TME, affecting the effectiveness of immunotherapy [172]. Specifically, apelin plays a role in the recruitment and activation of immune cells (i.e., macrophages and T cells) through the PI3K-Akt and ERK-MAPK pathways. *In vitro*, apelin released by tumor cells was found to promote M2 polarization of macrophages, which is strictly linked to immunosuppressive and tumor-promoting functions and could affect the efficacy of immunotherapy [128].

Additionally, apelin contributes to the shaping of TME by regulating angiogenesis and immune cell infiltration [173]. Apelin inhibition has also been shown to protect cancer patients from resistance to anti-angiogenic therapies and metastasization [74].

### 3.2. The Apelinergic System as a Risk Factor and Drug Resistance: Evidence from Experimental Models and Clinical Studies

In addition to a worse prognosis [16,174], the expression of the apelin/APJ system has been correlated with a worse response to drugs in different tumors, and has been proposed as a biomarker and predictor of therapy efficacy [174,175].

#### 3.2.1. Lung Cancer

In an *in vitro* model of lung carcinoma, overexpression of apelin-13 and APJ inhibited the cytotoxic effect of doxorubicin and razoxane by activating the PAK1/LIMK1/cofilin pathway and promoting cell invasion and migration. Conversely, APJ silencing re-established their antineoplastic effect [67]. In the same model, the authors demonstrated that hypoxia was able to increase apelin expression and to induce cancer progression [67].

In a mouse model, loss of apelin in combination with therapy with the VEGF-TKI sunitinib (40 mg/kg die) was shown to delay lung cancer growth [Lewis lung carcinoma 1]. Inhibition of apelin in combination with sunitinib greatly reduced the density of tumor vessels and decreased microvessel remodeling, demonstrating that apelin was a powerful pro-angiogenic signal supporting the onset of neovascularization [176]. According to these data, in apelin knockout mice the inhibition of apelin prevented resistance to anti-angiogenic RTK inhibitors, reducing tumor growth and angiogenesis without increasing hypoxia in the tumor microenvironment [74].

In a study involving 61 patients with NSCLC treated with one of three EGFR-TKIs (erlotinib, gefitinib, or icotinib), potential markers of therapy resistance were investigated using MALDI-TOF MS. Specifically, 25 patients with progressive disease or stable disease for ˃6 months were considered resistant to EGFR-TKIs, while 36 patients with partial response or stable disease for ˂6 months were judged sensitive to therapy. Notably, apelin expression was significantly increased in the EGFR-TKI-resistant group compared to the EGFR-TKI-sensitive group, suggesting that elevated apelin expression might be associated with EGFR-TKI resistance by influencing angiogenesis [177]. In contrast, a retrospective study that involved 81 patients with stage-IV NSCLC who received first-line therapy with platinum derivatives (cisplatin/carboplatin + gemcitabine) or second-line therapy with taxotere did not find a significant correlation between apelin levels and OS, PFS, chemoresistance, or treatment response (*p* ˃ 0.05) [178].

#### 3.2.2. Colon Cancer

The recurrence rate of colorectal cancer is high and resistance to anticancer drugs increases the treatment failure rate [179]. In this context, only 10–15% of patients benefit from the antiangiogenic drug bevacizumab (bvz) [180].

Zuurbier et al. identified apelin as a potential predictive biomarker in the response to bvz in patients with colorectal cancer, demonstrating high apelin levels in therapy-resistant patients with reduced PFS (high apelin: average 3.8 months, low apelin: average 11.06 months, *p* = 0.006) [180]. Zhang et al. evaluated the expression levels of apelin mRNA following a single administration of bvz in a xenograft model of colon adenocarcinoma. Apelin mRNA expression and plasma levels decreased transiently on the fifth day after treatment, accompanied by a significant reduction in tumor growth, blood vessel density and tumor hypoxia. Therefore, the authors proposed apelin as a potential indicator of the window of vessel normalization during antiangiogenic therapy [181]. HIF-1α plays a crucial role in radiation resistance of colorectal cancer. By next-generation RNA sequencing analysis of radio-sensitive and radio-resistant colon cancer cells exposed to normal and hypoxic conditions, apelin was demonstrated to be involved in HIF-1α-mediated cellular resistance to radiation [182].

#### 3.2.3. Gastric Cancer

The combination of endostar, an anti-angiogenic peptide, with chemoradiotherapy (CRT) is a therapeutic approach to GC, but, to date, no reliable markers have been able to predict the response to treatment and prognosis of these patients. In this view, apelin/APJ represents a promising tool. As a matter of fact, patients treated with CRT+endostar with high levels of APJ expression were 3.645 times more likely to have a poor response to the combined treatment than patients with low levels of APJ expression. Additionally, multivariate survival analysis showed that high APJ expression is an independent predictor of OS in patients treated with CRT+endostar [183].

#### 3.2.4. Hepatocellular Cancer (HCC)

Using a multi-omics approach, Song et al. showed that apelin/APJ was significantly overexpressed in the vascular endothelium of HCC, whereas its expression was lower in normal samples [77]. In particular, high apelin/APJ expression in endothelial cells was found to be associated with angiogenesis and tumor metastasis through the PI3K-Akt pathway and the apelin-HDAC5-KLF2 axis [77]. Pan-cancer analysis of single genes suggested that apelin may inhibit immune effector cells across various tumor types. In HCC tissues, the analysis of immune infiltration demonstrated significant immune suppression in the high-risk group (characterized by more progressed stages of disease and unfavorable prognosis), which implied non-responsiveness to immunotherapic drugs. In this scenario, apelin/APJ was identified as a crucial determinant of this immunosuppressive environment [77].

#### 3.2.5. Breast Cancer

A retrospective study that involved 62 patients with early-stage breast cancer treated with neoadjuvant chemotherapy (NAC), comprising taxanes and anthracyclines, revealed that, in addition to obesity [184], high tumor expression of the apelin/APJ system was associated with a reduced rate of pathological complete response (pCR) [38,185]. In turn, a low pCR resulted in worse PFS [186]. Notably, BMI and apelin have been described as two independent parameters associated with reduced response to NAC [185]. Furthermore, increased expression of the apelin/APJ system in tumor tissue was associated with increased tumor growth, decreased PFS and OS, increased microvessel density, lymphatic vessel density, and the presence of lymphatic metastases in breast cancer patients. This study showed that a combination therapy that inhibits both angiogenesis and lymphangiogenesis would probably be useful for these patients [20]. In contrast, Jinag et al. demonstrated that an overexpression of a gene pool, including apelin, significantly correlated with distant relapse-free survival (HR = 0.213, 95% CI [0.131–0.347], *p* = 4.80 × 10^−9^) in NAC-treated patients [187].

Dendritic cells are routinely applied to boost immune-based therapy against tumors, since they process tumor-specific antigens, express co-stimulatory molecules, produce cytokines, and migrate to lymphoid organs in order to present the antigens to specific T cells [188]. However, conventional immunotherapy methods, such as the dendritic cell vaccine, have been reported to be less effective in targeting cancers, due to the immunosuppressive microenvironment of the tumor. Given the importance of apelin/APJ in tumor progression, targeting this axis in combination with the dendritic cell vaccination may enhance anti-tumor immune responses. This hypothesis was confirmed by Masoumi et al., who treated a xenograft breast cancer model with the APJ antagonist ML221, a dendritic cell vaccine (DCV), or a combination of ML221 and DCV. Their results showed that single therapy with ML221 or DCV was able to reduce tumor growth (*p* < 0.0001), but the combination therapy had the greatest effect in reducing tumor size, preventing lung metastasis (*p* < 0.0001), and improving survival rates (*p* < 0.01) when compared to the control group. Additionally, ML221+DCV was the most effective in increasing Th1 cell and decreasing Th2 cell frequency in the spleen (*p* < 0.01) [189]. In a similar *in vivo* model, the combination therapy group also showed significantly decreased expression of MMP-2, MMP-9, CXCR4, VEGF, FGF-2, and TGF-β in tumor tissues (*p* < 0.05), as well as reduced serum levels of IL-9 and IL-35 (*p* < 0.0001) and vascular density and vessel diameter (*p* < 0.0001) [190]. In addition, apelin inhibition prevented resistance to the anti-angiogenetic RTK inhibitor treatment and reduced tumor progression *in vivo* [74].

#### 3.2.6. Glioblastoma

In apelin knockout mice, tumor growth was reduced and survival increased. Furthermore, the lack of apelin reduced the density of glioblastoma vessels below the normal levels observed in the striatum of a healthy mouse brain, emphasizing the transition to avascular tumor growth [81].

Glioblastoma’s resistance to chemotherapy is partly due to cancer stem cells. Harford-Wright et al. demonstrated that the APJ antagonist MM54 enhances cancer stem cells’ sensitivity to temozolomide (TMZ), suggesting that the MM54-TMZ combination can target chemotherapy-resistant cells. MM54 reduces GSC self-renewal and increases TMZ sensitivity by affecting the GSK3β pathway, which is upregulated in glioblastoma, promoting GSK3β phosphorylation and inhibiting its signaling [42]. Accordingly, Mastrella et al. evaluated the efficacy of anti-angiogenic therapy with bvz and its relationship with apelin and APJ expression both in *in vitro* and *in vivo* models. Their results showed that the apelin/APJ system plays a central role in resistance to bvz therapy. Indeed, bvz reduced apelin expression levels, but increased the invasive activity of APJ-expressing cells. Cell treatment with F13A, a mutant form of apelin that sequesters APJ, reduced angiogenic and invasive activity with synergistic effects to bvz, suggesting a possible clinical use of the combination therapy [65]. Regarding this aspect, a recent multi-omics analysis showed an altered expression of apelin after topotecan treatment and paclitaxel+topotecan combination treatment, indicating its role in the response to therapy [191].

#### 3.2.7. Chondrosarcoma

Surgical resection is the best choice for clinical treatment of chondrosarcoma, although high-grade chondrosarcoma is destructive and more likely to metastasize, making its surgical removal difficult [192]. To date, doxorubicin has been the most widely used chemotherapeutic agent, but the development of drug resistance makes treatment not always effective. In an *in vitro* study, Chen et al. verified high levels of apelin in the doxorubicin-resistant tumor cell line SW1353 compared to the parental tumor cell line. By array analysis on chondrosarcoma tissues, they also showed that apelin expression was higher in high-grade tissues than in low-grade tissues [193]. The downregulation of apelin through mir-631 resensitized SW1353 cells to doxorubicin treatment [193].

#### 3.2.8. Prostate Cancer

Li et al. observed an overexpression of APJ mRNA in various prostate cancer cell lines and in prostate cancer tissue samples. On the other hand, APJ silencing inhibited the intracellular PI3K/AKT/mTOR pathway, which is essential for cell proliferation, thereby promoting apoptosis and increasing the radiosensitivity of cancer cells [156].

#### 3.2.9. Ovarian Cancer

In patients affected by ovarian cancer and treated with bvz, increased apelin expression is associated with significantly reduced disease-free survival [174]. Accordingly, overexpression of apelin/APJ reduced the response of KOV3 ovarian cancer cells to anti-angiogenic therapy, leading to a reduced response to sorafenib treatment [194,195]. By using gene and protein profiling techniques, Jaiprasart et al. demonstrated that the apelin/APJ pathway plays a key role in resistance to the anti-angiogenic drugs bvz and sorafenib, and that high apelin expression correlates with a poor prognosis in patients treated with bvz [196].

## 4. Apelin/APJ System Inhibitors and Antagonists

Due to their roles in different physiological processes, the discovery of apelin and APJ inhibitors has opened up new scenarios in the treatment of several diseases, including heart failure, obesity, and cancer [64,175].

Apelin inhibition has been demonstrated to be effective in reducing some key characteristics of tumor progression. It was shown that silencing apelin with siRNA suppressed proliferation, invasion, and migration, and promoted cell apoptosis in an *in vitro* model of esophageal cancer [26]. In HCC, elevated APJ expression correlated positively with worse outcomes and non-response to immunotherapy, whereas apelin silencing reduced angiogenesis (*p* < 0.05), endothelial proliferation, expression of ANG2, KLF2, and VEGFA, and phosphorylation of ERK1/2 [77]. The synaptojanin-2 binding protein, which is able to reduce apelin gene expression and ERK1/2 phosphorylation, has been demonstrated to inhibit proliferation, migration, and angiogenesis of endothelial cells mediated by VEGF [197]. Recently, Chen D. et al. have shown that sempervirine, a natural alkaloid derived from traditional Chinese medicine with significant anticancer activities, acts by downregulating the apelin pathway [198]. Using the SKOV3 ovarian cell line, they demonstrated both *in vitro* and *in vivo* that this compound inhibits cell proliferation and invasion. Moreover, mRNA expression of CD34 was dose-dependently reduced by sempervirine treatment, with an effect comparable to that of 5-fluorouracil [198].

F13A is a molecule discovered in 2003 [199]. Even if it is unclear whether F13A is actually an APJ antagonist or an apelin agonist [200], Lee et al. verified that this compound counteracted the hypotensive effects of apelin-13 in hypertensive rats [201] and inhibited proliferation, migration, and survival of retinal Müller cells under hypoxic conditions in the opposite way to apelin [202]. Similarly, Piacault et al. verified that F13A significantly reduced the proliferation rate of colon adenocarcinoma cells at a concentration of 100 nM [50]. Subsequent studies have shown how treatment of cancer cells with F13A was able to significantly inhibit tumor growth, invasion, and angiogenesis in hepatocarcinoma cells [203], triple-negative breast cancer [204], ovarian cancer [194], and glioblastoma [65] cells. The use of molecular imaging to monitor tissue angiogenesis could be a major advantage in highlighting targeted pro- or anti-angiogenic therapy, depending on the clinical context [205]. Due to the role of the apelin/APJ system in promoting VEGF/VEGFR-mediated angiogenesis, an F13A-based PET radiotracer targeting APJ was recently developed for molecular imaging of angiogenesis. [68Ga]Ga-radiolabeled apelin-F13A (codenamed [68Ga]Ga-AP747) is able to quantify APJ expression and non-invasively monitor angiogenetic progression in tissues [205]. Since no radiotracers have been authorized for clinical practice to date, studying [68Ga]Ga-AP747 in more detail could provide a new approach for this clinical practice.

Since its discovery in 2012 [206], the 4-oxo-6-((pyrimidin-2-ylthio)methyl)-4H-pyran-3-yl 4-nitrobenzoate (ML221) APJ antagonist has been demonstrated to inhibit cancer progression and angiogenesis in different human cancer cell lines, such as cholangiocarcinoma [29], ovarian [32], HCC [22], choriocarcinoma [207], metastatic breast cancer [189], infantile hemangioma [208], and cervical [17] cancer cells, by modulating the PI3K/Akt pathway. In addition, it was shown that the immunotherapy+ML221 association may suppress and prevent lung metastasis in a metastatic breast cancer model, by reducing IL-10 and Th2 levels and increasing CD4+ and Th1 levels [189].

Protamine has also been identified as an inhibitor of APJ *in vitro* and in vivo, with a 390-nM affinity for APJ and a full antagonism of G protein- and β-arrestin-dependent intracellular signaling [209]. In both ex vivo and in vivo models, protamine abolished apelin-induced angiogenesis. Similarly, amodiaquine antagonized APJ and inhibited angiogenesis of HUVEC cells *in vitro* [210].

Co-administration of TMZ and the APJ antagonist MM54 has been shown to enhance sensitivity to therapy in glioblastoma cells both *in vitro* and in vivo by inhibiting the GSK3β signaling pathway [42]. In melanoma cells, APJ positively regulates IFN-γ signaling through the recruitment of β-arrestin 1, thus promoting tumor progression [117]. In a murine model of skin melanoma, MM54 administration (2 mg/kg) reduced tumor size (*p* = 0.0047), significantly inhibited the formation of blood vessels (*p* < 0.05), and decreased the number of proliferating cells in lung metastases (*p* < 0.0001) [211].

## 5. MiRNA Expression and the Apelin System in Cancer

Approximately 75% of the human genome is transcribed into RNAs, but only 3% into protein-coding mRNAs. Non-coding RNAs (ncRNAs) have been subdivided according to length, shape, and position into several classes: microRNA (miRNA), long ncRNA (lncRNA), circular RNA (circRNA), and PIWI-interacting RNA (piRNA), each with distinct functions in tumors as oncogenes or oncosuppressors [212,213].

There is increasing evidence of a bidirectional interaction between ncRNAs and apelin in different pathologies, including cancer. Numerous studies have shown a correlation between the apelinergic system and miRNA in patients with essential hypertension. In particular, apelin modulated the expression of miR-424 and miR-503, reducing the levels of FGF2 and its receptor FGFR1, which are involved in the proliferation of vascular smooth muscle cells. In this context, miR-15b-5p and miR-122-5p suppress apelin signaling by activating pro-inflammatory mediators such as interleukins and TNF-α, thus further aggravating hypertension [214,215,216]. Furthermore, microRNA-765 influences arterial stiffness by modulating apelin expression and inhibiting phosphorylation of eNOS and ERK/Akt/AMPK signaling [217]. In atherosclerosis, miR-497 is elevated and acts by downregulating *APLN* expression [218]. *APLN* expression protected neurons from death in epilepsy both *in vitro* and in vivo by inhibiting the expression of metabotropic glutamate receptor 1 (mGluR1), Bax, and caspase-3, promoting Bcl-2 expression and increasing pAKT levels. It has been shown that miR-182 negatively influenced this apelin neuroprotective effect [219]. Likewise, Elabela showed beneficial effects on neuronal survival after ischemia by inhibiting apoptosis and axonal damage through the increased expression of miR-124-3p [220]. In the pathologic processes of obesity and osteoarthritis, apelin stimulated the synthesis of IL-1β, a major pro-inflammatory cytokine, and inhibited the expression of miRNA-144-3p, which is able to block IL-1β transcription [221]. Again, apelin promoted angiogenesis by inhibiting miR-525-5p synthesis via PLCγ/PKCα signaling in rheumatoid arthritis pathogenesis, worsening this condition [222].

Regarding cancer, it has been shown that some of these ncRNAs, such as miR-224, miR-195/miR-195-5p, miR-204-5p, miR-631, miR-4286, miR-637, miR-4493, and miR-214-3p, target apelin mRNA and influence its expression in several tumor types. Circ-NOTCH1, circ-ZNF264, and lncRNA BACE1-AS also positively regulate apelin expression in the tumor context. On the other hand, apelin has also been shown to regulate the expression of some ncRNAs, such as circ_0000004/miR-1303, miR-15a-5p, and miR-106a-5p, thereby influencing tumorigenesis [172].

Previous findings showed that downregulation of miR-224 promoted PCa progression and was associated with poor biochemical recurrence-free survival [223]. In particular, TRIB1, which is overexpressed in various types of cancer, has been identified as a direct target of miR-224 [223]. In humans, it has been reported that this protein regulates the activation of several intracellular signaling pathways, including the mitogen-activated protein kinase pathway, promoting cell activation to apoptosis and modulation of gene expression in carcinoma cells [224]. Yueping et al. demonstrated that the overexpression of miR-224 reduced PCa cell invasion and migration by suppressing *APLN* expression, whereas lower miR-224 levels were associated with higher *APLN* mRNA expression in PCa tissues. Specifically, downregulation of miR-224 and apelin upregulation correlated with advanced clinical stage (*p* = 0.027) and metastasis (*p* = 0.001), suggesting that the disrupted miR-224/*APLN* axis played a key role in PCa tumorigenesis and aggressive progression [31]. On the other hand, *APLN* upregulation occurred more frequently in PCa tissues with advanced pathological stage (*p* = 0.003), presence of metastases (*p* < 0.001), and PSA failure (*p* = 0.001) [31]. Furthermore, PCa patients in the miR-224–low/*APLN*-high group more frequently had shorter biochemical recurrence-free survival than those in the groups with other expression patterns of the two molecules [31]. These findings suggest a strong association between the miR-224/*APLN* axis and tumorigenesis and aggressive progression of PCa. In contrast, evidence of elevated microRNA-224 expression and apelin levels was observed in colorectal cancer tissues, suggesting a potential role of this microRNA in the promotion of metastasis formation and poor patient survival outcomes and a positive association with the apelinergic system [225].

miR-195-5p regulates tumor cell proliferation, migration, invasion, and apoptosis through the modulation of different intracellular pathways. In particular, miR-195–5p inhibits tumor progression by negatively regulating the JAK/STAT3, TNF, and EGFA/Ras/Raf/MEK/ERK pathway. It also limits cell proliferation by targeting Notch, reduces tumorigenesis by blocking the Wnt/β-catenin pathway, represses cancer cell proliferation and invasion via YAP1–SEPT2–Hippo, and restricts tumor progression by reversing PI3K/AKT pathway activation [226]. Additionally, its expression may induce chemoresistance [226]. It has been shown that miR-195–5p enhances sensitivity to 5-fluorouracil and oxaliplatin in drug-resistant cells of gastric cancer by targeting ZNF139. Similarly, overexpression of miR-195–5p significantly reduced GDPD5 protein expression and the Notch signaling pathway, which are linked to glycolysis, metastases, and chemoresistance in colorectal cancer. In breast cancer cells, the expression of miR-195–5p was inversely associated with trastuzumab resistance. Furthermore, the overexpression of miR-195–5p downregulated angiogenesis and cisplatin resistance in ovarian cancer cells by targeting PSAT1 and inhibiting the GSK3β/β-catenin pathway [226]. The expression levels of apelin and miR-195 were analyzed in lung adenocarcinoma tissues and lung cancer cell lines, revealing an inverse correlation and a direct effect of miR-195 on apelin mRNA transcription. Overexpression of miR-195 significantly inhibited the proliferation, migration, and invasion of lung adenocarcinoma cells *in vitro* and suppressed tumor growth *in vivo*, suggesting its role as a possible therapeutic target [227]. In addition, it has been shown that miR-195-5p reduced apelin expression *in vitro* to inactivate the Wnt signaling pathway, inhibiting tumor invasiveness and angiogenesis [228].

miR-204-5p is frequently downregulated in various types of cancer and is associated with clinical-pathological features and the prognosis of cancer patients. To date, many studies have shown that miR-204-5p acts as a tumor suppressor due to its broad and powerful ability to inhibit tumor proliferation, metastasis, autophagy, and chemoresistance in multiple cancer types [229,230,231]. Both *in vitro* and *in vivo*, tumor-associated macrophages have been shown to promote chemoresistance in colorectal cancer. In particular, they act by promoting the IL6R/STAT3 pathway, which in turn inhibits the transcription of the tumor suppressor miR-204-5p [232]. In cisplatin-, etoposide-, and paclitaxel-resistant SCLC cells it was demonstrated that the lncRNA LYPLAL1-DT is upregulated, and its overexpression enhanced autophagy and inhibited apoptosis through miR-204-5 sponging [233]. MiR-204-5p was found to be poorly expressed in esophageal cancer tissues, while its target gene apelin was highly expressed. Additionally, overexpression of miR-204-5p inhibited the proliferation, invasion, migration, and stemness of esophageal cancer cells *in vitro*. Dual luciferase assay confirmed the interaction between miR-204-5p and apelin: miR-204-5p was able to reduce apelin levels, and its overexpression decreased the effects of apelin on cancer cell function. In view of these findings, the miR-204-5p/*APLN* pathway could serve as a potential target for the development of anticancer agents [234].

Likewise, miR-631 is also considered a tumor suppressor [235,236]. In bortezomib-resistant multiple myeloma cells, miR-631 was shown to be downregulated, while its overexpression enhanced the cell’s sensitivity to the drug [237]. Doxorubicin-resistant chondrosarcoma cells (SW1353 and JJ012) showed high apelin expression compared to parenteral cells and miR-631 was identified as the most likely regulator of apelin. Furthermore, the expression of miR-631 was observed to diminish in resistant cells; however, the ectopic expression of miR-631 in doxorubicin-resistant cell lines significantly increased doxorubicin sensitivity [193].

miR-4286 promotes cancer progression in various types of tumor [238,239,240] through modulating different pathways, such as JAK2/STAT3 [241], PI3K/Akt [242], and IGF-1/TGF-β [243]. For these reasons, it is considered an unfavorable prognostic marker in the cancer context. Inhibition of miR-4286 *in vitro* resulted in a 2.6-fold increase in the rate of apoptosis and a 1.7-fold decrease in cell proliferation/viability by altering the mRNA expression of several gene targets, including apelin [244].

circ-NOTCH1, a potential oncogene that confers drug resistance [245], was found to be overexpressed in gastric cancer tissues. In *in vitro* and *in vivo* models of gastric cancer, Zhao et al. demonstrated that the presence of circ-NOTCH1 promoted tumor growth and metastasis [246]. On the other hand, its suppression inhibited cell invasion and migration by downregulating the proto-oncogene MYC [246,247]. In addition, the overexpression of circ-NOTCH1 is correlated to resistance to doxorubicin therapy [245]. Studies on circ-NOTCH1 have indicated that miR-637 binds circ-NOTCH1 by also targeting apelin. In gastric cancer cell lines and tissues, circ-NOTCH1 and apelin are highly expressed, whereas miR-637 expression is reduced. *In vitro* tests on gastric cancer cell lines showed that circ-NOTCH1 positively regulated the expression of the target gene apelin, promoted increased cell proliferation and invasiveness, and reduced cell apoptosis, whereas miR-637 acted in the opposite way [248].

Analysis of circRNA expression in glioma cells showed that the expression of circ-ZNF264 was upregulated, whereas the expression of miR-4493 was inhibited. The dual luciferase reporter gene assay showed that miR-4493 could bind to circ-ZNF264 and apelin. Circ-ZNF264 has been shown to inhibit miR-4493 expression in these cells, promote apelin overexpression, and consequently positively regulate glioma cell proliferation, apoptosis, and invasion [249].

The role of lcnRNA BACE1-AS is controversial. It has been shown that its overexpression or downregulation can promote cell proliferation, invasion, and chemoresistance depending on the tumor type [250,251,252]. In 5-FU-resistant human colon cancer cell lines, the expression of BACE1-AS was downregulated [251]. Similarly, the expression of BACE1-AS decreased after the anisomycin treatment of human ovarian cancer stem cells, whereas its silencing resulted in drug resistance [250]. BACE1-AS also regulated cell proliferation and invasion in osteosarcoma cell lines and tissues in a negative manner [253]. In contrast, BACE1-AS was shown to be increased in HCC tissues and cell lines and it correlated with an increase in cell invasion and migration, through the miR-377-3p/CELF1 axis [252]. Tian and colleagues found that BACE1-AS expression was significantly higher in HCC cell lines, tissues, and patient serum, and its increased expression was linked to a poor prognosis. Overexpression of BACE1-AS enhanced cell proliferation, migration, invasion, and cell cycle progression, whereas it inhibited apoptosis. BACE1-AS also sponged miR-214-3p, reducing its expression and promoting apelin levels. The modulation of miR-214-3p partially reversed the effects of BACE1 manipulation, suggesting that the BACE1-AS/miR-214-3p/*APLN* axis plays a key role in HCC progression [254]. Moreover, in patients with HCC, the response to immunotherapy was correlated with BACE1-AS levels [255]. Similarly, *in vitro* and *in vivo* studies demonstrated that the overexpression of BACE1-AS is associated with an increase in metastatic colorectal cancer cells [256]. In patients, its overexpression is associated with a poor prognosis [256]. Wang et al. have proved that BACE1-AS acts via the miR-214-3p/TUFT1/Wnt signaling regulatory axis, essential for liver metastasis. In fact, the pharmacological inhibition of the Wnt signaling pathway has been shown to reduce liver metastasis and stem-like traits in CRC cells overexpressing BACE1-AS [256].

In tumors, increasing levels of the enzyme PLOD2 are associated with a poor outcome [70]. Trang et al. showed a positive correlation between apelin and PLOD2 levels in osteosarcoma tissues and cells, and this association is correlated with the presence of tumor metastasis. In particular, this effect is mediated by an upregulation of the Hyppo/YAP pathway and by a downregulation of circ_00004/miR-1303 [71].

miR-15a-5p has a double role in cancer progression. It acts as a tumor suppressor in HCC [257], neuroblastoma [258], and breast cancer [259] progression. In HCC cell lines, enhanced levels of miR-15a-5p expression suppressed migration, induced apoptosis, and caused G1-phase arrest. Accordingly, *in vivo* experiments confirmed the role of miR-15a-5p in HCC growth inhibition [260]. In endometrial cancer, it suppresses cell growth via the Wnt/β-catenin signaling pathway by inhibiting WNT3A [261]. Similarly, miR-15a-5p silences oncogenes, such as PDL1, FGFR1, DDX3X, SLC1A5, and FXR1, in NSCLC pathogenesis [262]. In contrast, miR-15a-5p behaves as an oncogene in renal cell carcinoma [263] and colorectal cancer progression, regulating the proto-oncogene MYB [264]. In gastric cancer cells, the overexpression of miR-15a-5p is associated with cisplatin resistance by promoting Akt phosphorylation [265]. Moreover, Vandewalle et al. demonstrated an overexpression of miR-15a-5p in acute myeloid leukemia patients who were non-responsive to chemotherapy with cytarabine and daunorubicin [266] by modulating the autophagy pathway [267]. miR-15a-5p promotes cell cycle arrest and sensitization of cells to doxorubicin in *in vitro* and *in vivo* models of bladder cancer through repressing EIFFA2, a factor that promotes EMT [268].

There is only one *in vitro* study that shows a correlation between the apelin/APJ system and miR-15a-5p. Specifically, apelin overexpression was associated with increased proliferation and invasive capacity of an NSCLC cell line and with a reduction in the exosomal miRNA miR-15a-5p, suggesting that miR-152-5p might have an anti-tumor role in lung cancer [269].

miR-106a-5p also has a dual role in cancer. In fact, it can show pro- [270,271] or anti-tumoral [272,273,274] effects, depending on the tumor. Additionally, it has been shown that colorectal cancer cells release exosomes enriched with miR-106a-5p, which promote M2 macrophage polarization by inhibiting SOCS6 and activating the JAK2/STAT3 pathway. In turn, M2 macrophages increase liver metastasis and the presence of miR-106a-5p is associated with a poor prognosis [275]. The expression of miR-106a-5p was associated with 5-fluorouracile resistance in an *in vitro* model of gastric cancer [276] and colorectal cancer [277], and with radio and TMZ resistance in glioma [278,279]. In contrast, in cisplatin-resistant osteosarcoma and lung cancer cells, miR-106a-5p was downregulated [280,281]. In PCa cells, apelin treatment promoted cell migration and invasion through inhibition of the tissue inhibitor of metalloproteinase 2 (TIMP2) expression. In turn, it has been demonstrated that overexpression of apelin promoted increased expression of miR-106a-5p via the c-Src/PI3K/Akt signaling cascades [282]. In PCa, miR-106a overexpression was also shown to confer radio-resistance by increasing proliferation and reducing senescence [283].

Given the pro- and anti-tumor roles of ncRNAs in cancer and the evidence linking these molecules to the apelin/APJ system, these findings suggest that their regulation, in conjunction with apelin and its receptor, could represent a promising new approach for molecular cancer diagnostics and the development of targeted therapeutic strategies.

The abovementioned evidence is summarized in Table 1.

## 6. Conclusions

Recent literature supports the crucial role of the apelinergic system in initiating and maintaining cancer growth by interacting at different levels with the complexity of cancer biology. In addition to promoting several hallmarks of cancer (“sustaining proliferative signaling”, “resisting cell death”, “activating invasion and metastasis”, “inducing/accessing vasculature”, “reprogramming cellular metabolism”, “avoiding immune destruction” and “tumor-promoting inflammation”, and “enabling replicative immortality”), the overexpression of apelin/APJ negatively influences tumor responses to drugs and immunotherapy. In this view, future studies will be essential to develop reliable and reproducible diagnostic and prognostic methods based on apelinergic hyperactivation in cancer patients, and to better clarify the possible use of therapeutic tools targeting apelin/APJ in clinical practice. The development of *in vitro* models (2D and 3D co-cultures, multicellular spheroids, multifluidic platforms) could help to elucidate the effects of apelin/APJ on cancer cells and the tumor microenvironment, in terms of secretion of pro-angiogenetic factors, endothelial function, activity of tumor-associated immune cells, immune checkpoint expression, and cytokine and chemokine production. Furthermore, APJ gene knockout animal models could shed new light on the pathophysiologic role of the apelinergic axis in tumorigenesis and on the putative role of apelin inhibition and APJ manipulation (e.g., CRISPR-based technologies, artificial intelligence) as anticancer agents, alone or in combination with other drugs, in order to potentiate their effect and/or reduce chemoresistance.

## Figures and Tables

**Table 1:** Evidence supporting the role of the apelinergic system in tumor resistance to anticancer therapies.

Effects of Apelin/APJ System in Cancer Patients and in Experimental Models of Cancer	
Modulation of Apelin/APJ and Impact on Prognosis	Resistance to Anticancer Treatments
***Lung cancer***	
Apelin expression in tissue positively correlates with more advanced disease and poorer outcomes in NSCLC [28] Apelin expression in tissue and circulating apelin positively correlate with a higher tumor stage, a higher probability of distant metastasis, and a shorter PFS and OS in lung adenocarcinoma [40] Apelin expression in tissue correlates with disease stage in lung cancer [43]	Resistance to TKIs [74,160,176,178] -increased apelin expression in EGFR-TKI-resistant patients compared to EGFR-TKI-sensitive subjects -inhibition of apelin in combination with sunitinib delays cancer growth and reduces the density of tumor vessels and microvessel remodeling Resistance to doxorubicin and razoxane [67] -overexpression of apelin-13 and APJ inhibits the cytotoxic effect of the drugs by activating the PAK1/LIMK1/cofilin pathway and promoting cell invasion and migration Resistance to cisplatin [233,281,282] -miR-106a-5p and miR-204-5 are downregulated in resistant lung cancer cells Resistant to etoposide and paclitaxel [233] -miR-204-5 is downregulated in resistant lung cancer cells Apelin expression in tissue predicts cancer progression independently of other potential confounders in patients affected by lung cancer and treated with one or more lines of chemotherapy, surgery, and/or radiotherapy [43]
***Colorectal cancer***	
Circulating apelin positively correlates with more advanced TNM stages, and lymph node and distant metastasis [33] Apelin expression in tissue correlates with disease stage [43]	Resistance to bevacizumab [180,181] -high apelin levels in bevacizumab-resistant patients with reduced progression-free survival -apelin mRNA expression and circulating levels decrease on the fifth day after bevacizumab treatment and are associated with reduction of tumor growth, blood vessel density, and tumor hypoxia Resistance to 5-fluorouracile [252,278] -expression of BACE1-AS is downregulated in resistant cells -expression of miR-106a-5p is associated with drug resistance Apelin expression in tissue predicts cancer progression independently of other potential confounders in patients affected by gastrointestinal cancer and treated with one or more lines of chemotherapy, surgery, and/or radiotherapy [43] Resistance to radiation [182] -apelin mediates HIF1α-dependent cellular resistance to radiation
***Gastric cancer***	
Apelin expression in tissue positively correlates with more advanced disease and poorer outcomes [27] Apelin expression in tissue correlates with disease stage in gastrointestinal cancer [43]	Resistance to endostar + chemiotherapy [183] -high levels of APJ expression in patients are associated with a poorer response (significantly shorter overall survival) to the combined treatment than patients with low APJ expression Resistance to 5-fluorouracil and oxaliplatin [226,277] -miR-195–5p enhances sensitivity in drug-resistant cells by targeting ZNF139 -expression of miR-106a-5p is associated with 5-fluorouracile resistance Resistance to doxorubicin [246] -overexpression of circ-NOTCH1 is correlated to resistance to doxorubicin Resistance to cisplatin [266] -overexpression of miR-15a-5p is associated with cisplatin resistance by promoting Akt phosphorylation Apelin expression in tissue correlates with disease stage and predicts cancer progression independently of other potential confounders in patients affected by gastrointestinal cancer and treated with one or more lines of chemotherapy, surgery, and/or radiotherapy [43]
***Esophageal cancer***	
Apelin expression in tissue positively correlates with a lower 3000-day survival rate [26]	
***Hepatocellular carcinoma (HCC)***	
Apelin expression in tissue: -is an independent prognostic factor of shorter overall survival [22] -is associated with a more rapid progression from T2 to advanced stages (T3 and T4) [24] APJ expression in tissue [23]: -is associated with the presence of intrahepatic metastasis, early recurrence, and shorter PFS and OS -is an independent predictor of shorter PFS	Resistance to immunotherapy [77,256] -apelin/APJ correlates with immune suppression in high-risk HCC and non-responsiveness to immunotherapic drugs -BACE1-AS levels directly correlate with response to immunotherapy
***Cholangiocarcinoma***	
Apelin expression in tissue positively correlates with more advanced disease and poorer outcomes [29]	
***Breast cancer***	
Apelin expression in tissue: -significantly correlates with tumor size and stage, microvessel density, lymph node metastasis, and worse disease-free and overall survival [20,38] -is an independent predictor of HER-2/neu expression and a more aggressive breast cancer phenotype [21] -correlates with disease stage and predicts cancer progression independently of other potential confounders in patients affected by gastrointestinal cancer and treated with one or more lines of chemotherapy, surgery, and/or radiotherapy [43] -together with Snail, is positively associated with lymph node metastases and TNM staging, thus correlating with a poor prognosis [159] Circulating apelin positively correlates with a higher tumor stage, a higher probability of distant metastasis, and a shorter PFS and OS [38]	Resistance to chemotherapy [38,185,186] -high tumor expression of apelin/APJ is associated with a reduced rate of pathological complete response to chemotherapy and a worse progression-free survival Resistance to trastuzumab [226] -expression of miR-195–5p was inversely associated with drug resistance Resistance to immunotherapy [189,190] -combined therapy with the APJ antagonist ML221 and a dendritic cell vaccine (DCV) is more effective than single therapies in reducing tumor growth, vascular density and vessel diameter, preventing lung metastases, and improving survival rates through an increase of Th1/Th2 ratio in the spleen Resistance to RTK inhibitors [74] -apelin inhibition prevents resistance to the anti-angiogenetic RTK inhibitors treatment and reduced tumor progression in vivo
***Glioblastoma/glioma***	
APJ expression in tissue is associated with advanced stage and worse prognosis [34] Apelin expression in tissue and circulating apelin positively correlate with a higher tumor stage, a higher probability of distant metastases, and a shorter PFS and OS [41,42] Increased expression of Elabela is associated with poor prognosis [45]	Resistance to bevacizumab [65] -bevacizumab reduces apelin expression levels but increases the invasive activity of APJ-expressing cells -treatment with a mutant form of apelin that sequesters APJ reduces angiogenic and invasive activity with synergistic effects to bevacizumab Resistance to temozolomide [42,279,280] -APJ antagonist MM54 enhances cancer stem cell sensitivity to the drug by affecting the GSK3β pathway -expression of miR-106a-5p is associated with radio and temozolomide resistance Resistance to topotecan [191] -apelin expression is altered after topotecan treatment and paclitaxel+topotecan combination ttherapy
***Head and neck cancer***	
Apelin expression in tissue and circulating apelin positively correlate with a higher tumor stage, a higher probability of distant metastasis, and a shorter PFS and OS [36,37]	
***Prostate cancer***	
Apelin upregulation in tissue more frequently occurs in prostate cancer tissues with advanced tumor stage and metastasis and is associated with a shorter biochemical recurrence-free survival [31] Apelin expression in tissue correlates with disease stage [43]	*APLN* silencing enhances the radiosensitivity of prostate cancer by downregulating the PI3K/Akt/mTOR pathway and the Bcl-2 protein family, and by promoting apoptosis mediated by Bax and caspase-3 [156] Apelin expression in tissue predicts cancer progression independently of other potential confounders in patients treated with one or more lines of chemotherapy, surgery, and/or radiotherapy [43]
***Muscle-invasive bladder cancer***	
Apelin expression in tissue and circulating apelin positively correlate with a higher tumor stage, a higher probability of distant metastasis, and a shorter PFS and OS [35]	Resistance to doxorubicin [269] -miR-15a-5p promotes sensitization of cells to doxorubicin
***Chondrosarcoma***	
	Resistance to doxorubicin [193] -apelin is more expressed in doxorubicin-resistant tumor cells -apelin expression is higher in high-grade cancer tissues than in low-grade ones -miR-631 is a key regulator of apelin expression in doxorubicin-resistant cells
***Multiple myeloma***	
Apelin expression in tissue and circulating apelin positively correlate with a higher tumor stage, a higher probability of distant metastasis, and a shorter PFS and OS [39]	Resistance to doxorubicin [193] -apelin is more expressed in doxorubicin-resistant tumor cells -apelin expression is higher in high-grade cancer tissues than in low-grade ones -miR-631 is a key regulator of apelin expression in doxorubicin-resistant cells Resistance to bortexomib [237] -miR-631 is downregulated in resistant cells
***Ovarian cancer***	
APJ expression in tissue significantly correlates with a decreased OS (14.7 months) in patients with high-grade cancer [32] Apelin expression in tissue correlates with disease stage [43]	Resistance to bevacizumab [174,196] -increased apelin expression is associated with reduced disease-free survival and a poor prognosis Resistance to sorafenib [194,195] -overexpression of apelin/APJ reduces the response of cancer cells to sorafenib Resistance to cisplatin [226] -overexpression of miR-195–5p downregulates cisplatin resistance by targeting PSAT1 and inhibiting the GSK3β/β-catenin pathway Resistance to anisomycin [251] -BACE1-AS silencing results in drug resistance Apelin expression in tissue predicts cancer progression independently of other potential confounders in patients treated with one or more lines of chemotherapy, surgery, and/or radiotherapy [43]
***Cervical cancer***	
Apelin expression in tissue positively correlates with advanced clinicopathologic features, poor therapy outcomes, and short survival [17,18,19] Apelin expression in tissue correlates with disease stage [43]	Resistance to bevacizumab [174] -increased apelin expression is associated with reduced disease-free survival Resistance to sorafenib [194,195] -overexpression of apelin/APJ reduces the response of cancer cells to sorafenib Apelin expression in tissue predicts cancer progression independently of other potential confounders in patients treated with one or more lines of chemotherapy, surgery, and/or radiotherapy [43]

HIF1α: hypoxia-inducible factor 1α.
